# Co-morbidity of malnutrition with falciparum malaria parasitaemia among children under the aged 6–59 months in Somalia: a geostatistical analysis

**DOI:** 10.1186/s40249-018-0449-9

**Published:** 2018-07-06

**Authors:** Damaris K. Kinyoki, Grainne M. Moloney, Olalekan A. Uthman, Elijah O. Odundo, Ngianga-Bakwin Kandala, Abdisalan M. Noor, Robert W. Snow, James A. Berkley

**Affiliations:** 1 Spatial Health Metrics Group, INFORM Project, Kenya Medical Research Institute/Wellcome Trust Research Programme, Nairobi, Kenya; 2 Nutrition Section, United Nations Children’s Fund (UNICEF), Kenya Country Office, UN Complex Gigiri, Nairobi, Kenya; 3 Warwick Medical School, Health Sciences Research Institute, Warwick Evidence, University of Warwick, Gibbet Hill, Coventry, CV4 7AL UK; 4 Food Security and Nutrition Analysis Unit (FSNAU) - Somalia, Food and Agriculture Organization of the United Nations, Ngecha Road Campus, Nairobi, Kenya; 5 Department of Mathematics and Information sciences, Faculty of Engineering and Environment, Northumbria University, Newcastle upon Tyne, UK; 6 Faculty of Health and Sport Sciences, University of Agder, Kristiansand, Norway; 7 Division of Epidemiology and Biostatistics, School of Public Health, University of the Witwatersrand, Johannesburg, South Africa; 8 Kenya Medical Research Institute/ Wellcome Trust Research Programme, Centre for Geographic Medicine Research (coast), Kilifi, Kenya; 9 Centre for Tropical Medicine and Global Health, Nuffield Department of Clinical Medicine, University of Oxford, CCVTM, Oxford, OX3 7LJ UK

**Keywords:** Malnutrition, Wasting, Low-MUAC, Malaria, Comorbidity, Somalia

## Abstract

**Background:**

Malnutrition and malaria are both significant causes of morbidity and mortality in African children. However, the extent of their spatial comorbidity remains unexplored and an understanding of their spatial correlation structure would inform improvement of integrated interventions. We aimed to determine the spatial correlation between both wasting and low mid upper arm circumference (MUAC) and falciparum malaria among Somalian children aged 6–59 months.

**Methods:**

Data were from 49 227 children living in 888 villages between 2007 to 2010. We developed a Bayesian geostatistical shared component model in order to determine the common spatial distributions of wasting and falciparum malaria; and low-MUAC and falciparum malaria at 1 × 1 km spatial resolution.

**Results:**

The empirical correlations with malaria were 0.16 and 0.23 for wasting and low-MUAC respectively. Shared spatial residual effects were statistically significant for both wasting and low-MUAC. The posterior spatial relative risk was highest for low-MUAC and malaria (range: 0.19 to 5.40) and relatively lower between wasting and malaria (range: 0.11 to 3.55). Hotspots for both wasting and low-MUAC with malaria occurred in the South Central region in Somalia.

**Conclusions:**

The findings demonstrate a relationship between nutritional status and falciparum malaria parasitaemia, and support the use of the relatively simpler MUAC measurement in surveys. Shared spatial distribution and distinct hotspots present opportunities for targeted seasonal chemoprophylaxis and other forms of malaria prevention integrated within nutrition programmes.

**Electronic supplementary material:**

The online version of this article (10.1186/s40249-018-0449-9) contains supplementary material, which is available to authorized users.

## Multilingual abstracts

Please see Additional file 1 for translations of the abstract into the five official working languages of the United Nations.

## Background

Infection and nutrition are intimately related through shared pathways involving poverty, limited national capacities for prevention, and effects on metabolism and immunity [1]. Distinguishing the relative contributions of infectious diseases and nutrition as causes of death is complex as most childhood deaths due to undernutrition are ultimately caused by infections rather than starvation. Thus, in national reporting systems and estimates of the global burden of diseases, infectious diseases are presented an immediate, direct cause of death. Malnutrition may only be recognized as the cause of death when it is severe enough to cause clinical manifestations and be classified as severe acute malnutrition (SAM) [2]. Pelletier et al. first demonstrated that malnutrition caused more than half of child mortality through its synergistic relationship with common infections, a much larger proportion than deaths classified under “nutritional deficiencies” [2]. Similarly, community-based studies of malaria demonstrate that its contribution to under-fives mortality is much greater than can be attributed to malaria-specific deaths alone [2]. Both malaria and undernutrition are highly prevalent in sub-Saharan Africa, where child mortality remains above international targets [3–5].

The relationship between undernutrition and malaria is not well understood, and has been a subject of competing hypotheses. Nutritional interventions have been observed to worsen the outcome of malaria episodes among children in Nigeria [6–9] and Senegal [7]. This suggested to some authors that iron deficiency may protect against malaria [10]. However, others have found no evidence to support the hypothesis that one or more forms of under-nutrition protect against malaria and its severity [11–14]. In fact, in several studies, an increased risk of poor outcomes of malaria have been described in the context of malnutrition [13] suggesting that malnutrition and malaria form a vicious circle with its predominant impact on vulnerable populations and likely operating via a range of effects on functional immunity [4, 15].

In Somalia, the southern region has a prevalence of acute malnutrition of at least 35% [16] and the distribution of malaria has a strikingly similar pattern [17]. The *Plasmodium falciparum* parasite rate (P*f*PR) has an estimated range of 0–52%, with higher P*f*PR locations occurring in the more highly populated regions between the Juba and Shabelle rivers. The dryer northern part of Somalia has a reported P*f*PR of less than 5% [18, 19].

Several pathways may explain the co-occurrence of these two conditions. On one hand, children are at risk of both malnutrition and infections due to their living environment, and thus prone to concurrent conditions occurring by chance [20]. Both conditions are subject to the same seasonal variations driven by weather and agricultural food supply. On the other hand, malnutrition is known to compromise immunity to infection, although mechanisms are unclear [21]. In return, malaria causes anorexia, weight-loss, low consumption of nutrients on generating inflammatory responses, iron deficiency, and in pregnant women, causes low birth weight.

The overlapping epidemiology of these health conditions may be explored by joint mapping to determine the correlation structures between their common, and disease-specific effects, as well as spatial and seasonal patterns simultaneously [22]. In this study, we undertook a nationwide investigation of the ecological co-morbidity of two forms of malnutrition, wasting and low mid upper arm circumference (MUAC), and falciparum malaria in Somalia. Common and indicator-specific unobserved and unmeasured spatial risks were fitted using a shared component model [23, 24].

### Country context

Somalia is mostly semi-arid with more arid areas in the north and central regions. There are four main seasons: *gu* is the predominant rainy season April–June; *Hagaa* is a dry season July–September; *Deyr* is a shorter rainy season October–November; and *Jillal* is the longer dry season December–March.

Most Somalis depend on pastoralism and agro-pastoralism. Pastoralists largely live in the arid rural areas, including around Somalia’s borders with Kenya and Ethiopia. Agro-pastoralist communities compete for local water and farmland resources [25]. A small proportion of the population in the centre and south of Somalia undertakes settled agriculture close to the two permanent rivers, Juba and Shabelle. Despite Somalia having one of the longest coastlines in Africa, fishing represents only a very limited livelihood activity [25].

In 1991, the national government’s collapse in 1998 led to the emergence of autonomous zones in the northern part of the country, the “Republic of Somaliland” and the “Puntland State of Somalia”. In the south and central regions a military administration was established in 1999. However, the autonomy of these zones is not internationally recognized [26]. The social, economic and public health infrastructure has been overwhelmed by chronic conflict that has resulted in massive internal and external population displacement and environmental degradation. Consequently, this has severely affected human development across the country [27].

Multiple United Nations (UN) agencies and Non-governmental Organization (NGO) partners came together to form a ‘nutrition cluster’ in 2006. In this way environmental, food security and nutritional status surveillance was established, aiming to provide information to improve the timing and effectiveness of nutritional and health responses. Large-scale assistance programs are ongoing in attempt to avert crises, however their coverage and ability to deliver interventions is hampered by insecurity, the harsh climate and the weak public health system [27].

## Methods

### Survey data

**Fig. 1:**
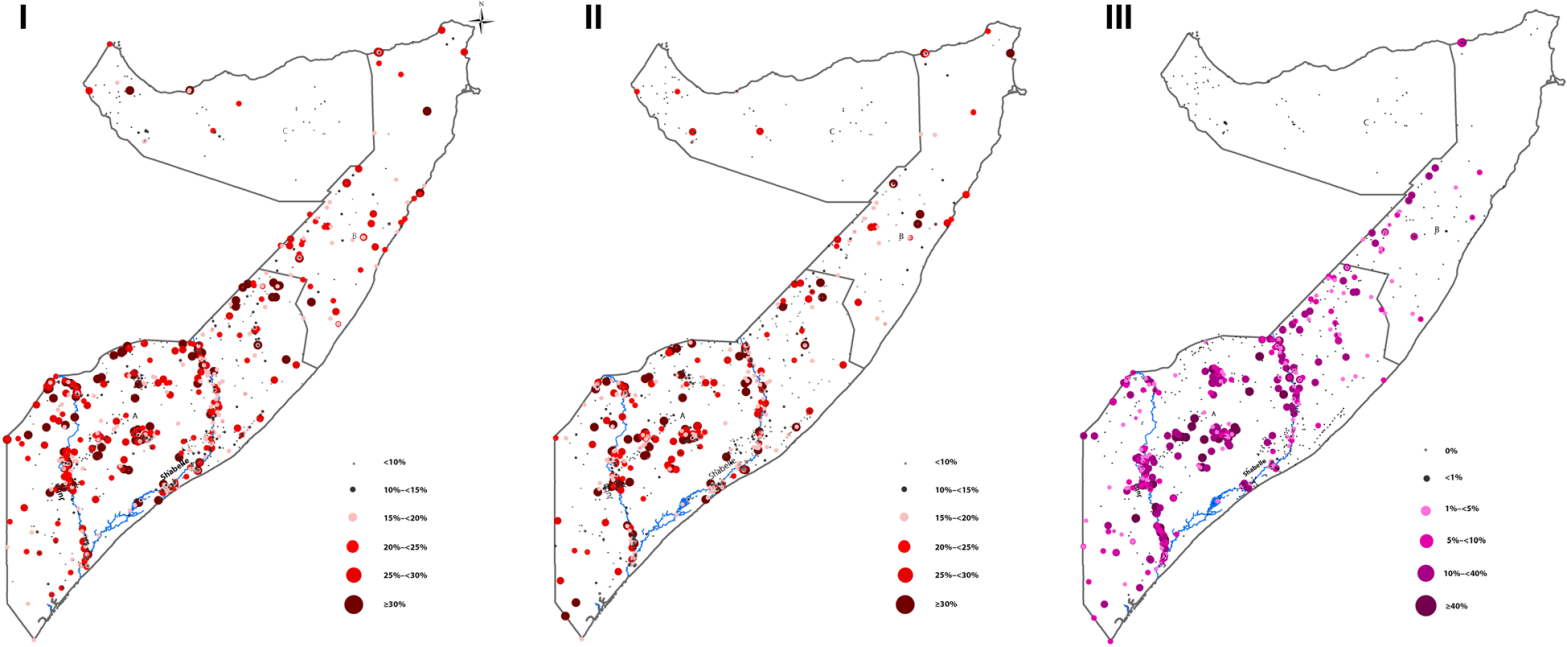
Map showing the distribution of the observed prevalence of (I) = wasting, (II) = low-MUAC and (III) = malaria in villages sampled for FSNAU nutrition surveys conducted between 2007 and 2010 in Somalia. The country is divided into three main zones: A = South Central B = North East, C = North West. 48 villages were sampled in North West zone, 85 villages in the North East zone and 755 villages in the South Central. The country’s two main rivers, Juba and Shebelle are located in the South Central zone. **MUAC:** Mid-Upper arm circumference; **FSNAU**: Food security and nutrition analysis unit

The Food Security and Nutrition Unit (FSNAU) was established in 1994 to monitor food security, malnutrition and livelihoods in Somalia to meet the needs of both emergency responses and longer-term planning. FSNAU, together with United Nations International Children’s Emergency Fund (UNICEF), conducted bi-annual surveys of population nutritional status and health between 2007 and 2010 (Fig. 1). The surveys included assessment of falciparum malaria parasitaemia and were therefore utilized for this study [26, 28].

A stratified multi-stage cluster sampling design was used for the surveys, with a sampling frame by district, livelihoods (pastoral, agro-pastoral, riverine and fishing) and urban/rural location. Internally displaced persons (IDPs) were surveyed separately. Villages were selected from a prior list at a probability proportional to their population sizes. In each of 30 villages, 30 households were then randomly selected, as previously described [28, 29]. Where the number of households in the village was unknown, it was estimated from the population figures then, starting from a random household, every n^th^ household was selected. Survey items included household size, age structure, gender of the household head, and 24-h recall of access to and intake of foods. Data on deaths were collected from selected households, including those without children aged 6–59 months.

All children aged 6–59 months living in selected households were measured (Additional file 2: Fig. S1). Childrens’ gender, age, weight, height, mid-upper arm circumference (MUAC) were measured. History of polio and measles immunization, vitamin A supplementation in the last six months, as well as diarrhea, acute respiratory infections (ARI) and febrile illness in the prior two weeks were collected. Data on falciparum malaria infection in children aged 5–59 months were collected in sub-sets of villages at the request of UNICEF [28–30]. The data used in this study were therefore a subset of the whole survey dataset with information on both the childhood malnutrition and malaria. The spatial coordinates of each village were determined, and remotely sensed environmental data were extracted, as previously described [29–31].

We investigated two anthropometric indicators of malnutrition, low weight-for-height (wasting) and low MUAC. These indicators generally detect only partially overlapping sets of children as malnourished. Wasting is the traditional indicator used in community surveys, however MUAC is quicker to perform and a better predictor of mortality [32]. Wasting is defined as <− 2 *Z* scores for weight-for-height, according to World Health Organization (WHO) 2006 growth references [33]. MUAC < 125 mm was classified as low-MUAC. Malaria parasitaemia was determined using Paracheck Pf™ (Orchid Biomedical Systems, Goa, India) rapid diagnostic test in a subset in every FSNAU surveys during this period [33]. A child was classified as malaria-infected when s/he had a positive Paracheck Pf™ test result for falciparum malaria, regardless of clinical symptoms.

### Statistical methods

The overall aim of this study was to model the ecological comorbidity of wasting and low MUAC with malaria parasitaemia among children aged 6–59 month in Somalia from 2007 to 2010. To achieve this, we developed a Bayesian geostatistical shared component model through a stochastic partial differential equation (SPDE) approach with integrated nested Laplace approximations (INLA) using the R-INLA library in R project version 3.2.3 [21–23, 34, 35]. We modelled the underlying spatial risks common to: (1) wasting and malaria, and (2) low-MUAC and malaria at the child level. Predictors from the surveys and environmental predictors of malnutrition and malaria were controlled at individual, household and village level. Relative risks were determined from the latent spatial component shared by each pair of conditions, and a condition-specific component after controlling for the environmental covariates [36, 37].

Finally, in order to determine if risks were spatially correlated, we performed a significant test examining the 2.5 and 97.5% quantiles of each element of the random effect using the quintile correction (QC) method as implemented by Bolin and Lindgren 2012 [38]. The empirical correlation between the conditions was also explored using correlation plots. Detailed methods on covariate selection, geostatistical shared component modelling, and the validation procedures are described elsewhere [29].

## Results

A total of 49 227 children aged 5–59 months, with a mean age of 32 months (51% male) were examined from 888 villages. Of which 8542 (17%), 5276 (11%) and 6840 (14%) were wasted, had low-MUAC and malaria parasitaemia respectively (Fig. 1 and Additional file 2: Fig. S2). Fever in the last two weeks was reported for 21% of children, while 26 and 17% reported symptoms consistent with acute respiratory infection (ARI) and diarrhoea respectively. Approximately, 97, 87 and 79% of children were reported to have consumed sources of carbohydrate, protein or fats in the last 24 h before the survey respectively. Fifty-seven percent had received Vitamin A supplementation in the two weeks prior to the survey and 51 and 82% reported to have received measles and polio vaccination respectively. A summary of individual level data is shown in Table 1.

**Table 1:** Baseline characteristics of the study population

Characteristic		Number
Total number of children examined		49 227
Total number of villages examined		888
Summary by livelihood		Number (%)
Livelihood	Agro-pastoral	14 018 (28)
	Pastoral	14 190 (29)
	Riverine	9618 (20)
	Fishing	335 (1)
	Urban areas	2769 (6)
	Internally Displaced Persons	8297 (17)
Response variables		Number (%)
Wasting		8542 (17)
Low-MUAC		5276 (11)
Malaria		6840 (14)
Child data		Number (%)
Vitamin A supplementation		28 264 (57)
Measles vaccination		26 184 (51)
Polio vaccination		39 309 (82)
Diarrhoea		9517 (17)
Acute Respiratory Infection (ARI)		10 493 (26)
Febrile Illness in the last 2 weeks		10 409 (21)
Suspected measles in last 1 month		2171 (5)
Sex of the child		Male = 25 067 (51)
Age of the child (in months)		Mean = 32, Min = 6, Max = 59
Household data		Mean (Min, Max)
Household size		6 (2, 50)
Number of under 5		2 (1, 7)
Age of the mother (in years)		30 (14, 60)
MUAC of mother in cm		24 (18, 38)
Household head gender		Male = 40 076 (81%)
Food and nutrition		Number (%)
Sources of carbohydrate in the last 24 h		47 560 (97)
Sources of protein in the last 24 h		42 713 (87)
Sources of fats in the last 24 h		39 130 (79)
Fruits and vegetables in the last 24 h		20 895 (42)
Climatic / Environmental data		Mean (Min, Max)
Distance to water to major water bodies in km		≤5 km = 18 445 (25%), > 5 km = 55 333 (75%)
Enhanced Vegetation Index (EVI)		0.18 (0, 0.45)
Precipitation (mm)		138 (0, 350)
Temperature (°c)		28 (21, 31)
Urbanization		Urban = 3318 (5%) Rural = 70 460 (95%)

By livelihood, 28, 29 and 20% of children were from areas of agro-pastoral, pastoral and riverine livelihoods respectively, while 17% lived in internally displaced people (IDP) camps and 6% lived in urban areas. The mean household size was 6 with a median of 2 in children 5–59 months. Eighty one percent of the household had male head.

**Fig. 2:**
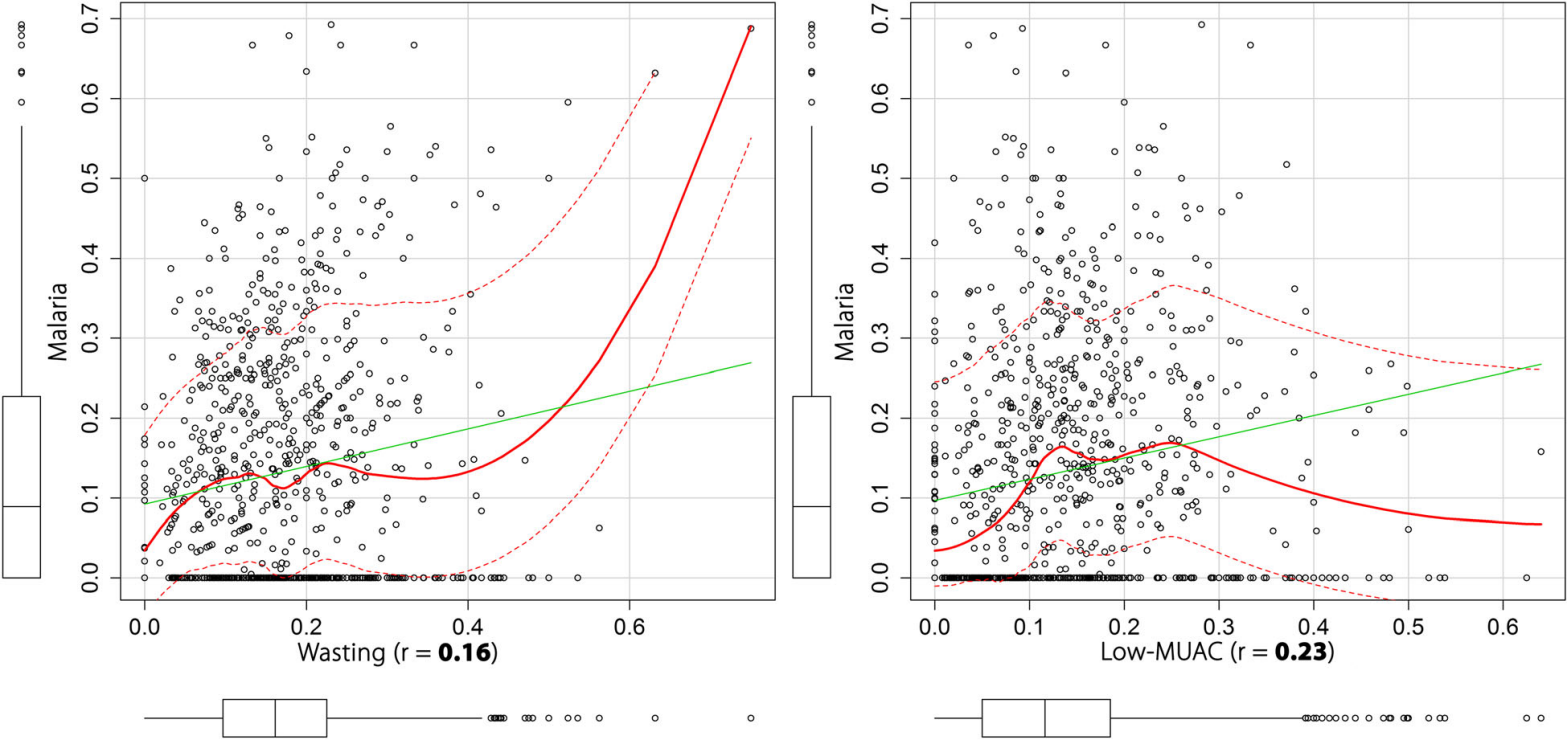
Correlation plots of wasting and low-MUAC with malaria among children under the age of five years in Somalia. MUAC: Mid-Upper arm circumference

**Fig. 3:**
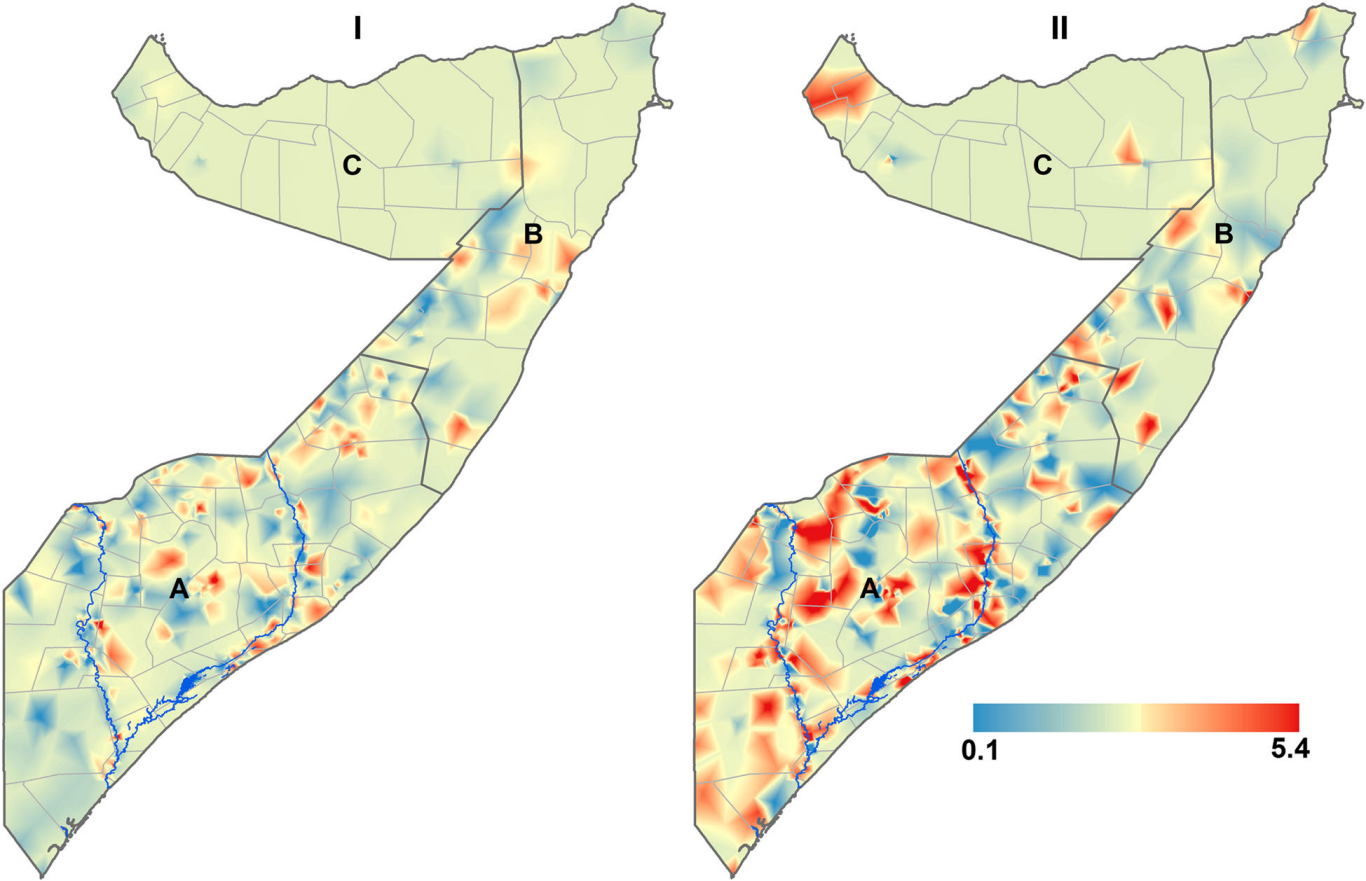
The predicted 1 × 1 km posterior maps showing the shared relative risk between (I) Wasting and malaria (II) Low-MUAC and malaria among children aged 6–59 months in Somalia. A = South Central zone, B = North East (Puntland) zone, C = North West (Somaliland) zone. MUAC: Mid-Upper arm circumference

The correlation between empirical estimates was highest between malaria infection and low-MUAC at 0.23, and relatively lower between malaria and wasting at 0.16, as shown in Fig. 2. As a first step, the associations of child-level, household and environmental covariates with wasting and low-MUAC were examined in a univariate and multiple variable binomial regression analysis. The effects of the covariates can be found in Additional file 2: Table S1 in the supplementary information file. The shared spatial residual effects were significant for wasting and malaria and for low-MUAC and malaria: odds ratio (*OR*) = 1.06, 95% credible interval (CrI): 1.04–1.09 and *OR* = 2.74, 95% CrI: 2.38–3.14 respectively. The common spatial effects from the geostatistical shared latent component analysis are shown in Fig. 3. There was a strong spatial gradient in the South-North direction in all the shared components examined in this study. The range of relative risks between wasting and malaria was 0.11 to 3.55, and between low-MUAC and malaria was 0.19–5.40. In the South central region, hotspots were consistently found in Bakool, Bay and Shabelle Dhexe for both the wasting and malaria and the low-MUAC and malaria components, while in the North, hotspots were found in Nugaal and Awdal for the low-MUAC and malaria component only.

Table 2 gives the effects of the covariates controlled in the joint model. ARI and febrile illnesses were associated with high risk of wasting, low-MUAC and malaria. Consumption of food high in carbohydrates and proteins was associated with lower risk of wasting, low-MUAC and malaria. Precipitation and EVI were associated with lower risk of wasting (*OR* = 0.94, 95% CrI: 0.91–0.97); *OR* = 0.67, 95% CrI: 0.65–0.69 and low-MUAC (*OR* = 0.91, 95% CrI: 0.87–0.94; *OR* = 0.96, 95% CrI: 0.92–0.99), but increased risk of malaria (*OR* = 1.20, 95% CrI: 1.14–1.25; *OR* = 1.27, 95% CrI: 1.21–1.33). The ambient temperature was associated with higher risk of the wasting (*OR* = 1.15, 95% CrI: 1.11–1.19) and low-MUAC (*OR* = 1.17, 95% CrI: 1.12–1.24), and lower risk of malaria (*OR* = 0.80, 95% CrI: 0.75–0.86). Urbanization was associated with lower risk of malaria (*OR* = 0.60, 95% CrI: 0.53–0.68) but was not associated with wasting or low-MUAC. Children who slept under bed nets had a lower risk of malaria (*OR* = 0.83, 95% CrI: 0.79–0.87).

**Table 2:** Estimated regression coefficients (Odds ratio and 95% credible interval (Crl)) for the posterior marginal density for the covariates used in the shared component model

Correlates		Wasting		Low-MUAC		Malaria	
		Odds ratio	95% CrI	Odds ratio	95% CrI	Odds ratio	95% CrI
Individual data							
Vitamin A supplementation		1.00	(0.97–1.03)	0.98	(0.94–1.02)	**0.93**	**(0.89–0.98)**
Measles vaccination		1.01	(0.97–1.04)	1.00	(0.96–1.04)	**0.94**	**(0.89–0.99)**
Polio vaccination		0.94	(0.92–0.97)	1.01	(0.97–1.05)	**0.95**	**(0.90–0.99)**
Diarrhoea		**1.11**	**(1.08–1.14)**	**1.25**	**(1.21–1.30)**	1.02	(0.97–1.07)
Acute Respiratory Infection (ARI)		**1.04**	**(1.01–1.07)**	**1.07**	**(1.04–1.11)**	**1.21**	**(1.16–1.27)**
Febrile Illness		**1.04**	**(1.01–1.07)**	**1.08**	**(1.04–1.12)**	**1.14**	**(1.09–1.20)**
Suspected measles		1.02	(0.99–1.04)	1.02	(0.99–1.06)	1.00	(0.96–1.04)
Sex of the child (Female)		**0.85**	**(0.83–0.87)**	**1.11**	**(1.07–1.14)**	1.02	(0.98–1.06)
Child age (< 12 months as reference)	12–23 months	**0.91**	**(0.89–0.93)**	**0.86**	**(0.84–0.88)**	**1.09**	**(1.07–1.11)**
	24–59 months	**0.80**	**(0.73–0.87)**	**0.86**	**(0.79–0.93)**	0.91	(0.83–1.00)
Household data							
Household size		**1.04**	**(1.01–1.07)**	**1.13**	**(1.09–1.18)**	0.96	(0.92–1.00)
Number of under5		1.00	(0.98–1.03)	**1.05**	**(1.01–1.09)**	**1.07**	**(1.02–1.11)**
Female household head		0.99	(0.97–1.02)	0.97	(0.94–1.00)	0.96	(0.92–1.00)
Age of the mother (20–30 years as reference)	< 20 years	1.02	(1.00–1.05)	1.01	(0.98–1.05)	0.96	(0.92–1.00)
	31–40 years	**0.94**	**(0.91–0.97)**	**0.85**	**(0.76–0.95)**	**0.92**	**(0.88–0.96)**
	41–50 years	0.92	(0.79–1.06)	0.89	(0.78–1.02)	0.99	(0.95–1.03)
	> 50 years	1.03	(0.97–1.09)	0.74	(0.47–1.16)	0.98	(0.94–1.03)
MUAC of mother		**0.90**	**(0.88–0.92)**	**0.90**	**(0.87–0.93)**	0.97	(0.93–1.01)
Food access data							
High carbohydrate foods		**0.95**	**(0.93–0.98)**	**0.93**	**(0.90–0.97)**	**0.86**	**(0.81–0.91)**
High protein foods		**0.95**	**(0.92–0.97)**	**0.94**	**(0.91–0.98)**	**0.93**	**(0.89–0.97)**
Fats		0.99	(0.97–1.02)	**0.93**	**(0.90–0.97)**	**0.94**	**(0.90–0.98)**
Fruits and vegetables		0.97	(0.95–1.00)	0.96	(0.93–1.00)	1.03	(0.98–1.07)
Village data							
Enhanced Vegetation Index (EVI)		**0.67**	**(0.65–0.69)**	**0.96**	**(0.92–0.99)**	**1.27**	**(1.21–1.33)**
Rainfall		**0.94**	**(0.91–0.97)**	**0.91**	**(0.87–0.94)**	**1.20**	**(1.14–1.25)**
Temperature		**1.15**	**(1.11–1.19)**	**1.17**	**(1.12–1.24)**	**0.80**	**(0.75–0.86)**
Urbanization		1.00	(0.97–1.04)	0.91	(0.78–1.07)	**0.60**	**(0.53–0.68)**
Child slept under the net		0.98	(0.95–1.00)	0.99	(0.95–1.03)	**0.83**	**(0.79–0.87)**

Bold values are significant

## Discussion

The main objective of this study was to investigate the nationwide spatial comorbidity between wasting and low-MUAC with malaria. To achieve this, we implemented two geostatistical shared component methods to model the comorbidity between (1) wasting and malaria, and (2) low-MUAC and malaria at child level. The findings showed a strong co-occurrence of these health conditions. The relative risk was higher between low-MUAC and malaria than between wasting and malaria. The common risks were greater in the South than in the North of Somalia.

Numerous studies have investigated the epidemiological relationship between child malnutrition with either malaria disease or intensity of infection [13, 14, 39, 40]. In contrast, only a few studies have examined the spatial underlying component [41, 42]. This is the first study that has modelled the co-distribution of wasting and low-MUAC with malaria in a geostatistical framework on a national scale to produce continuous maps of common relative risks at high spatial resolution. The shared component statistical framework has the advantage that its latent components have a direct interpretation in terms of the prevalence of comorbidity and related risk factors, which are either shared by several or specific to one of the health conditions.

This study provides important new information about subnational priority areas for targeting integrated interventions for malnutrition and malaria. Our predictive maps of the common relative risk indicate that integrated control programs should be prioritized in the south of Somalia. The hotspots correlate with areas where malaria risk has been shown in previous studies [18, 19]. The hotspots therefore present opportunities for integrating malaria interventions with the nutrition interventions delivered through health campaigns by World Food Programme (WFP) and UNICEF which include vitamin A distribution, deworming and nutritional screening during bi-annual ‘child health days’ with a full course of antimalarial treatment during the peak malaria season which coincides with peak malnutrition levels [43]. Importantly, such seasonal malaria chemoprevention has been shown to be 75% protective against both uncomplicated and severe malaria in children [44–47] and may be effective in this setting.

The study has some limitations. There may be potentially important socio-demographic and environmental confounding factors that were not measured and therefore not accounted for in the analysis. For example, information on access to water and sanitation was not collected in the FSNAU surveys. In addition, information on market prices, purchasing power, food distribution, and household economic status that might influence household food security were not available and therefore not included in the analysis.

## Conclusions

There are significant spatial correlations between both wasting and low-MUAC and falciparum malaria in children aged 6–59 months in Somalia, indicating common underlying determinants. The findings support the use of MUAC to detect multiple co-morbidity risks and reinforce the need for integrating malaria and nutrition interventions.

## Additional files

Additional file 1: Multilingual abstracts in the five official working languages of the United Nations. (PDF 193 kb)

Additional file 2: **Table S1.** Univariate and multiple variable regression adjusted odds ratio (AOR) and 95% credible interval (CrI) of the effect of covariates on wasting and low-muac among children aged 6–59 months in Somalia. Values in bold typeface are those that don’t contain the value 1 in their 95% CrI and were considered statistically significant. **Fig. S1.** Flowchart for FSNAU surveys. This diagram was adopted from the ‘Guidelines for emergency nutrition and mortality surveys in Somalia’. The sample size of acute malnutrition and malaria are computed separately depending on the estimated prevalence and the desired precision but the sampling procedure is the same. **Fig. S2.** Patterns of stunting among children under the age of five in Somalia. These data were obtained from Food Security and Nutrition Unit (FSNAU) surveys ranging from the year 2007 to 2010. (DOCX 358 kb)

## Abbreviations

ARI: Acute respiratory infections
CrI: Credible interval
EVI: Enhanced vegetation index
FAO: Food and Agriculture Organization
FSNAU: Food security and nutrition analysis unit
GRUMP: Global Rural Urban Mapping Project
INLA: Integrated nested laplace approximations
MODIS: MODerate-resolution imaging spectroradiometer
MUAC: Mid-Upper arm circumference
NGO: Non-governmental Ogranization
*OR*: Odds ratio
PPS: Probability proportional to size
QC: Quintile correction
SMC: Seasonal malaria chemoprevention
SPDE: Stochastic partial differential eq.
UN: United Nations
UNICEF: United Nations Children’s Emergency Fund
WFP: World Food Programme
WHO: World Health Organization
