# Supplementary material for: Co-morbidity of malnutrition with falciparum malaria parasitaemia among children under the aged 6–59 months in Somalia: a geostatistical analysis
Source: Infect Dis Poverty. 2018 Jul 6;7:72. doi: 10.1186/s40249-018-0449-9 (PMC6036667; doi:10.1186/s40249-018-0449-9)
Supplement: Supplementary file 1 — Multilingual abstracts in the five official working languages of the United Nations. (PDF 193 kb) [file 40249_2018_449_MOESM1_ESM.pdf]

Translation of the abstract into the five official working languages of the United Nations

انتشار حالات سوء التغذية المصحوبة بالملاريا المنجلية الطفيلية بين الأطفال الذين هم دون سن 6-59 شهور في الصومال : تحليل احصائي جغرافي

قدمه: داماريس كينيوكي، وغرينن مولينى، وأولاليكان عثمان، وإيليا أودوندو، وإنجيانغا باكون كاندالا، وعبد العزيز محمد نور، وروبرت سنو وجيمس بيركلي

الملخص
المعلومات الأساسية: يعتبرُ سوء التغذية والملاريا على حد سواء من الأسباب الرئيسة للإصابة بالأمراض والوفيات بين الأطفال الأفريقيين. مع ذلك، ما زال معدل الاعتلال المكاني غير مستكشف بعد وكما أن التوصل إلى فهم البنية المكانية المترابطة سيسترشدُ به في تحسين التدخلات المتكاملة. نهدفُ إلى تحديد الترابط المكاني بين كلاً من الهزال ومقياس محيط العضد المتدني والملارياالمنجلية بين الأطفال الذين تتراوح أعمارهم بين 6-59 شهور.
الأساليب: جُمعت البيانات من عدد 49،777 من الأطفال يعيشون في 888 قرية بين عام 2007 إلى عام 2010. طورنا نموذج عنصر مشترك للإحصاء الجغرافي على نهج بايزي بغرض تحديد التوزيعات المكانية المشتركة بين الهزال و مرض الملاريا المنجلية; ومقياس محيط العضد المتدني و الملاريا المنجلية باستبانة مكانية تصلُ إلى 1 × 1 كيلو متر.
النتائج: يُستنتج أن هناك ارتباطات عملية للملاريا بنسبة 0.16 و 0.23 على التوالي للهزال ومقياس محيط العضد المتدني. إن الأثار المشتركة للبقايا المكانية ذات دلالة إحصائية مهمة لكلٍ من الهزال ومقياس محيط العضد المتدني. سُجلت نسبة الخطر المكاني بأعلاها لتدني مقياس محيط العضد والملاريا حيث تتراوح من (0.19 إلى 5.40) و تنخفض نسبياً بين الهزال والملاريا حيث تتراوح من (0.11 إلى 3.55). تقع المناطق الساخنة المشتركة لكلٍ من الهزال وتدني مقياس محيط العضد المصحوب بالملاريا في المنطقة الجنوبية الوسطى في الصومال.
الاستنتاجات: تُظهرُ النتائج وجود علاقة مشتركة بين حالة التغذية والملاريا المنلجية الطفيلية، ودعم استخدام قياسات بسيطة نسبياً لحساب مقياس محيط العضد في الدراسات الاستقصائية. تتيحُ المشاركة بين التوزيع المكاني والمناطق الساخنة المختلفة فرصاً موجهة للوقاية الكيميائية من الملاريا الموسمية وغيرها من أشكال المرض مدمجة ضمن برامج التغذية.

Translated from English version into Arabic by Dana muhb and Bashaier Allam, through

TRANSLATORS
WITHOUT BORDERS

索马里 6-59 个月儿童共患营养不良和恶性疟寄生虫血症的地理统计分析

Damaris K. Kinyoki, Grainne M. Moloney, Olalekan A. Uthman, Elijah O. Odundo, Ngianga-Bakwin Kandala, Abdisalan M. Noor, Robert W. Snow and James A. Berkley

摘要：
引言：营养不良和疟疾是造成非洲儿童发病和死亡的重要原因。然而，它们的共患病空间特征仍未被探索，了解其空间相关性结构将会改进综合干预措施。本研究旨在确定索马里 6-59 个月儿童消瘦和低中上臂围（MUAC）与恶性疟之间的空间相关性。
方法：本研究统计了索马里 2007 至 2010 年间 888 个村庄的 49,227 名儿童的数据。我们开发了贝叶斯地理统计共享组件模型，以确定消瘦与恶性疟、低 MUAC 与恶性疟的共同空间分布（1×1 km 空间分辨率）。
结果：消瘦和低 MUAC 与疟疾的经验相关值分别是 0.16 和 0.23。共享空间残差效应对消瘦和低 MUAC 均具有统计学意义。低 MUAC 与疟疾的后空间相对风险最高（值域：0.19–5.40），而消瘦与疟疾的后空间相对风险较低（值域：0.11 至 3.55）。索马里中南部地区是消瘦、低 MUAC 和疟疾的高发区。
结论：本研究结果表明了营养状况与恶性疟寄生虫血症存在相关性，它也支持在调查中使用相对简易的 MUAC 测量方法。共享空间分布和明显的热点地区有助于将定向季节性化学预防和其他形式疟疾预防纳入营养计划。

Translated from English version into Chinese by Qing-Yun Chen, edited by Jin Chen

Analyse géostatistique de la comorbidité due à la malnutrition et au *falciparum* malaria parasitaemia chez les enfants âgés de 6 à 59 mois en Somalie

Damaris K. Kinyoki, Grainne M. Moloney, Olalekan A. Uthman, Elijah O. Odundo, Ngianga-Bakwin Kandala, Abdisalan M. Noor, Robert W. Snow et James A. Berkley

Résumé

Contexte: La malnutrition et la malaria sont deux causes importantes de morbidité et de mortalité chez les enfants africains. Cependant, l'étendue de leur comorbidité territoriale demeure inexplorée et une meilleure compréhension de leur structure de corrélation territoriale permettrait d'améliorer les interventions intégrées. Nous voulions déterminer la corrélation territoriale entre le poids-pour-taille et le périmètre brachial (PB) et *falciparum* la malaria chez les enfants somaliens âgés de 6 à 59 mois.

Méthodes: Les données provenaient de 49 227 enfants vivant dans 888 villages différents. Elles ont été recueillies entre 2007 et 2010. Nous avons développé un modèle bayésien de composant partagé géostatistique afin de déterminer la distribution territoriale commune du poids-pour-taille et *falciparum* malaria ; et le PB de la partie inférieure du bras et *falciparum* malaria dans la résolution spatiale de 1 x 1 km.

Résultats: Les corrélations empiriques avec la malaria étaient respectivement de 0.16 et 0.23 pour le poids-pour-taille et pour le PB de la partie inférieure du bras. Les effets résiduels territoriaux communs avaient une importance sur le plan statistique pour le poids-pour-taille ainsi que pour le PB de la partie inférieure du bras. À posteriori, le risque relatif territorial était plus élevé pour le PB de la partie inférieure du bras et la malaria (de 0,19 à 5,40) et relativement plus bas pour le poids-pour-taille et la malaria (de 0,11 à 3,55). Les zones à risque pour le poids-pour-taille mais aussi pour le PB de la partie inférieure du bras avec la malaria se trouvent dans les régions du centre et du sud de la Somalie.

Conclusions : Les résultats démontrent le lien existant entre l'état nutritionnel et *falciparum* malaria parasitaemia. Cela confirme que l'on peut continuer à utiliser une mesure relativement plus simple du PB dans les études. Une distribution territoriale commune et différentes zones à risques présentent des possibilités de chimioprophylaxie ciblée et saisonnière. Cela permet aussi d'utiliser d'autres formes de prévention de la malaria, comme celles qui font partie des programmes de nutrition.

Translated from English version into French by Valérie Ourset and WillSquire, through

TRANSLATORS
WITHOUT BORDERS

Недоедание как сопутствующий фактор при заболевании *тропической* малярией с наличием паразитов в крови среди детей в возрасте от 6 до 59 месяцев в Сомали: геостатистический анализ

Дамарис К. Кинёки, Грайн М. Молони, Олалекан А. Усман, Элижах О. Одундо, Нгянга-Баквин Кандала, Абдисалан М. Нур, Роберт У. Сноу и Джеймс А. Беркли

Аннотация

Введение: Недоедание совместно с малярией является серьезной причиной заболеваемости и смертности детей в Африке. Однако, степень их территориальной совмещённости остаётся не исследованной и понимание структуры их территориальной корреляции скажется на улучшении комплексных мер по оперативному исправлению ситуации. Мы поставили своей целью определение территориальной корреляции между истощением и *тропической* малярией и измерениями окружности середины плеча (ОСП) и тропической малярией среди детей в возрасте от 6 месяцев до 59 месяцев в Сомали.

Методика: Данные были получены от 49 тысяч 277 детей, живущих в 888 деревнях в период между 2007 и 2010 годами. Мы использовали Байесовскую геостатистическую модель групповых компонентов, для того чтобы определить среднестатистическое территориальное распределение между истощением совместно с *тропической* малярией и низкими значениями ОСП совместно с *тропической* малярией с территориальным разрешением 1 км. на 1 км.
Результаты:Экспериментальная корреляция между малярией и истощением, и малярией и низкими значениями ОСП была соответственно 0.16 и 0.23. Групповые территориальные остаточные результаты были статистически значительными как при использовании метода определения степени истощения, так и при использовании метода регистрации низких значений ОСП. Относительный остаточный территориальный риск был выше при методе, использующем регистрацию низких значений ОСП совместно с малярией (диапазон от 0.19 до 5.40) и относительно меньшим при методе, использующем регистрацию истощения совместно с малярией (диапазон от 0.11 до 3.55). Проблемные места как при методе, использующем определение степени истощения, так и при методе, использующем регистрацию низких значений ОСП были найдены в центре южного региона Сомали.
Выводы:Полученные данные показывают связь между качеством питания и *тропической* малярией с паразитами в крови, и обосновывают использование относительно более простого способа использующего измерения ОСП при исследованиях. Групповое территориальное распределение и четко выраженные проблемные места обеспечивают возможность использования целевой сезонной химиопрофилактики и других методов предупреждения малярии, объединённых с программами питания.

Translated from English version into Russian by akiselyov and Ekaterina_Rugg, through

TRANSLATORS
WITHOUT BORDERS

Comorbilidad de la desnutrición por parasitemia ocasionada por malaria *falciparum* en niños entre 6 y 59 meses en Somalia: un análisis geoestadístico

Damaris K. Kinyoki, Grainne M. Moloney, Olalekan A. Uthman, Elijah O. Odundo, Ngianga-Bakwin Kandala, Abdisalan M. Noor, Robert W. Snow y James A. Berkley

Resumen

Antecedentes: La desnutrición y la malaria son causas importantes de morbilidad y mortalidad entre los niños africanos. Sin embargo, el alcance de su comorbilidad espacial sigue sin explorarse, y la comprensión de su estructura de correlación espacial informaría una mejora de las intervenciones integradas. Quisimos determinar la correlación espacial entre emaciación y circunferencia del brazo (MUAC) y malaria *falciparum* entre los niños somalíes de 6 a 59 meses de edad.
Métodos: Los datos corresponden a 49,227 niños que vivieron en 888 aldeas entre 2007 y 2010. Se desarrolló un modelo geoestadístico bayesiano de componente compartido para determinar las distribuciones espaciales comunes de emaciación y malaria *falciparum*; y de bajo contenido de MUAC y malaria *falciparum* a 1 × 1 km de resolución espacial.
Resultados: Las correlaciones empíricas con la malaria fueron de 0.6 y 0.23 para emaciación y 0.23 para MUAC bajo, respectivamente. Los efectos espaciales residuales compartidos fueron estadísticamente significativos tanto para la emaciación como para MUAC bajo. El riesgo relativo espacial posterior fue más alto para MUAC bajo y malaria (rango: 0.19 a 5.40) y relativamente más bajo entre emaciación y malaria (rango: 0.11 a 3.55). En la región centro meridional de Somalia se registraron focos de emaciación y de MUAC bajo con malaria.
Conclusiones: Los hallazgos demuestran una relación entre el estado nutricional y parasitemia por malaria *falciparum*, y apoyan el uso de la medición relativamente más simple de MUAC en las encuestas. La distribución espacial compartida y los focos distintos ofrecen oportunidades para la

quimioprofilaxis estacional selectiva y otras formas de prevención de la malaria integradas en los programas de nutrición.

Translated from English version into Spanish by Reina X. Sanjurjo and Noris La Valle, through

TRANSLATORS
WITHOUT BORDERS
