# Supplementary material for: Co-morbidity of malnutrition with falciparum malaria parasitaemia among children under the aged 6–59 months in Somalia: a geostatistical analysis
Source: Infect Dis Poverty. 2018 Jul 6;7:72. doi: 10.1186/s40249-018-0449-9 (PMC6036667; doi:10.1186/s40249-018-0449-9)
Supplement: Supplementary file 2 — Table S1. Univariate and multiple variable regression adjusted odds ratio (AOR) and 95% credible interval (CrI) of the effect of covariates on wasting and low-muac among children aged 6–59 months in Somalia. Values in bold typeface are those that don’t contain the value 1 in their 95% CrI and were considered statistically significant. Fig. S1. Flowchart for FSNAU surveys. This diagram was adopted from the ‘Guidelines for emergency nutrition and mortality surveys in Somalia’. The sample size of acute malnutrition and malaria are computed separately depending on the estimated prevalence and the desired precision but the sampling procedure is the same. Fig. S2. Patterns of stunting among children under the age of five in Somalia. These data were obtained from Food Security and Nutrition Unit (FSNAU) surveys ranging from the year 2007 to 2010. (DOCX 358 kb) [file 40249_2018_449_MOESM2_ESM.docx]

***Co-morbidity of malnutrition with malaria parasitaemia among children under the age of five years in Somalia: a geostatistical analysis***

***Supplementary Information: Model output***

**Table SI 1:** Univariate and multiple variable regression adjusted odds ratio (AOR) and 95% credible interval (CrI) of the effect of covariates on wasting and low-muac among children aged 6 – 59 months in Somalia. Values in bold typeface are those that don’t contain the value 1 in their 95% CrI and were considered statistically significant.

| **Covariates** | |  |  |  |  |  |  |  |  |  |
| --- | --- | --- | --- | --- | --- | --- | --- | --- | --- | --- |
|  |  | **Wasting** | | | | | **Low-MUAC** | | | |
|  |  | **Bivariate** | | **Multiple variable with interaction terms** | | | **Bivariate** | | **Multiple variable with interaction terms** | |
|  |  | **Odds ratio** | **CrI** | **Odds ratio** | | **CrI** | **Odds ratio** | **CrI** | **Odds ratio** | **CrI** |
| **Child data** | |  |  |  |  | |  |  |  |  |
| Malaria | | **1.33** | **(1.25,1.41)** | **1.26** | **(1.18,1.35)** | | **1.41** | **(1.31,1.52)** | **1.19** | **(1.10,1.30)** |
| Vitamin A supplementation | | 0.99 | (0.94,1.03) | 0.99 | (0.93,1.05) | | **0.84** | **(0.79,0.89)** | 0.94 | (0.87,1.02) |
| Measles vaccination | | 1.00 | (0.96,1.05) | 1.01 | (0.95,1.07) | | **0.87** | **(0.82,0.92)** | 1.01 | (0.93,1.09) |
| Polio vaccination | | **1.11** | **(1.05,1.18)** | **0.89** | **(0.83,0.96)** | | **0.83** | **(0.77,0.89)** | 0.97 | (0.89,1.06) |
| Diarrhoea | | **1.38** | **(1.30,1.46)** | **1.32** | **(1.24,1.40)** | | **2.15** | **(2.01,2.29)** | **1.74** | **(1.62,1.87)** |
| Acute Respiratory Infection (ARI) | | **1.21** | **(1.14,1.28)** | **1.12** | **(1.05,1.18)** | | **1.42** | **(1.32,1.52)** | **1.18** | **(1.10,1.28)** |
| Febrile Illness | | **1.23** | **(1.16,1.30)** | **1.12** | **(1.06,1.19)** | | **1.45** | **(1.35,1.55)** | **1.22** | **(1.13,1.31)** |
| Suspected measles | | **1.19** | **(1.06,1.32)** | 1.04 | (0.93,1.16) | | **1.26** | **(1.10,1.44)** | 1.02 | (0.89,1.18) |
| Sex of the child (Female) | | **0.73** | **(0.69,0.76)** | **0.73** | **(0.69,0.76)** | | **1.21** | **(1.14,1.28)** | **1.22** | **(1.15,1.30)** |
| Chid age (< 12 reference) | 12 - < 24 months | 0.94 | (0.89,1.00) | **0.89** | **(0.84,0.95)** | | **3.24** | **(3.03,3.46)** | **3.05** | **(2.85,3.27)** |
|  | 24 – 59 months | **1.14** | **(1.05,1.24)** | 1.07 | (0.98,1.17) | | **4.64** | **(4.25,5.05)** | **4.37** | **(3.99,4.79)** |
| **Household data** | |  |  |  |  | |  |  |  |  |
| Household size | | 0.99 | (0.98,1.00) | 0.99 | (0.98,1.00) | | **0.93** | **(0.92,0.95)** | **0.95** | **(0.94,0.97)** |
| Number of under5 | | **0.97** | **(0.94,0.99)** | 0.99 | (0.96,1.02) | | **0.91** | **(0.88,0.94)** | 1.04 | (1.00,1.08) |
| Female household head | | 1.01 | (0.96,1.08) | 0.99 | (0.93,1.05) | | **0.90** | **(0.83,0.98)** | **0.91** | **(0.84,0.99)** |
| Age of the mother | | 1.00 | (0.99,1.00) | 1.00 | (1.00,1.00) | | **0.99** | **(0.98,0.99)** | 1.00 | (1.00,1.01) |
| MUAC of mother | | **0.99** | **(0.98,0.99)** | **0.99** | **(0.98,0.99)** | | **0.99** | **(0.98,0.99)** | **0.99** | **(0.98,0.99)** |
| **Food and nutrition** | |  |  |  |  | |  |  |  |  |
| Carbohydrate | | **0.95** | **(0.91,0.99)** | 1.02 | (0.97,1.07) | | **0.84** | **(0.8,0.89)** | 1.00 | (0.94,1.06) |
| Protein | | **0.90** | **(0.88,0.92)** | **0.91** | **(0.88,0.94)** | | **0.89** | **(0.86,0.91)** | **0.91** | **(0.88,0.94)** |
| Fats | | **0.92** | **(0.86,0.97)** | 1.03 | (0.97,1.10) | | **0.77** | **(0.72,0.83)** | 0.93 | (0.85,1.01) |
| Fruits and vegetables | | **0.93** | **(0.90,0.96)** | 0.98 | (0.94,1.01) | | **0.95** | **(0.91,0.99)** | 1.00 | (0.95,1.05) |
| **Climatic / Environmental data** | |  |  |  |  | |  |  |  |  |
| Season (Deyr as reference) | Gu | 1.05 | (1.00,1.10) | 1.01 | (0.96,1.07) | | 0.99 | (0.93,1.05) | **0.91** | **(0.84,0.98)** |
|  | Hagaa | **0.62** | **(0.49,0.78)** | **1.37** | **(1.05,1.78)** | | **0.41** | **(0.29,0.59)** | 1.26 | (0.84,1.89) |
|  | Jilaal | **1.19** | **(1.07,1.31)** | **1.13** | **(1.00,1.28)** | | **1.17** | **(1.03,1.32)** | **2.33** | **(1.98,2.73)** |
| Distance to water | | 1.00 | (1.00,1.00) | 1.00 | (1.00,1.00) | | 1.00 | (1.00,1.00) | 1.00 | (1.00,1.00) |
| Enhanced Vegetation Index (EVI) | | **1.66** | **(1.23,2.24)** | **1.62** | **(1.16,2.27)** | | **2.64** | **(1.81,3.86)** | **2.70** | **(1.73,4.20)** |
| Rainfall | | 1.00 | (1.00,1.00) | 1.00 | (1.00,1.00) | | 1.01 | (1.00,1.01) | 1.01 | (1.01,1.01) |
| Temperature | | **1.12** | **(1.10,1.13)** | **1.13** | **(1.11,1.16)** | | **1.18** | **(1.16,1.21)** | **1.17** | **(1.14,1.21)** |
| Urbanization | | **1.27** | **(1.15,1.41)** | **0.81** | **(0.71,0.92)** | | **1.72** | **(1.53,1.93)** | **1.78** | **(1.52,2.08)** |
|  | |  |  |  |  | |  |  |  |  |
| **Interaction terms** | |  |  |  |  | |  |  |  |  |
| Carbohydrate : Distance to water | |  |  | 1.00 | (1.00,1.00) | |  |  | 1.00 | (1.00,1.00) |
| Carbohydrate: EVI | |  |  | 0.74 | (0.36,1.52) | |  |  | 0.65 | (0.24,1.72) |
| Carbohydrate : Precipitation | |  |  | 1.00 | (0.99,1.00) | |  |  | 1.00 | (1.00,1.01) |
| Carbohydrate: Temperature | |  |  | 0.97 | (0.94,1.01) | |  |  | 0.95 | (0.90,1.00) |
| Carbohydrate : Urbanization | |  |  | 1.14 | (0.91,1.44) | |  |  | **1.41** | **(1.05,1.89)** |
| Protein : Distance to water | |  |  | 1.00 | (1.00,1.00) | |  |  | 1.00 | (1.00,1.00) |
| Protein : EVI | |  |  | **1.91** | **(1.28,2.83)** | |  |  | 0.85 | (0.49,1.45) |
| Protein: Precipitation | |  |  | 1.00 | (1.00,1.00) | |  |  | 1.00 | (1.00,1.00) |
| Protein: Temperature | |  |  | 0.99 | (0.97,1.01) | |  |  | 1.01 | (0.98,1.04) |
| Protein: Urbanization | |  |  | 1.14 | (0.97,1.32) | |  |  | **1.22** | **(1.01,1.49)** |
| Distance to water : EVI | |  |  | 1.00 | (0.99,1.00) | |  |  | 1.01 | (1.00,1.01) |
| Distance to water: Precipitation | |  |  | 1.00 | (1.00,1.00) | |  |  | 1.00 | (1.00,1.00) |
| Distance to water : Temperature | |  |  | 1.00 | (1.00,1.00) | |  |  | 1.00 | (1.00,1.00) |
| Distance to water: Urbanization | |  |  | 1.00 | (0.99,1.00) | |  |  | 1.00 | (1.00,1.01) |
| EVI : Precipitation | |  |  | 1.00 | (0.98,1.02) | |  |  | **1.04** | **(1.02,1.07)** |
| EVI : Temperature | |  |  | 0.76 | (0.54,1.08) | |  |  | **2.06** | **(1.27,3.36)** |
| EVI : Urbanization | |  |  | 0.29 | (0.03,2.47) | |  |  | 0.02 | (0.00,0.30) |
| Precipitation : Temperature | |  |  | 1.00 | (1.00,1.00) | |  |  | 1.00 | (1.00,1.01) |
| Precipitation : Urbanization | |  |  | 0.99 | (0.97,1.01) | |  |  | 0.99 | (0.96,1.02) |
| Temperature : Urbanization | |  |  | 0.92 | (0.70,1.21) | |  |  | 1.26 | (0.86,1.85) |

MUAC: Mid-Upper Arm Circumference; EVI: Enhanced Vegetation Index


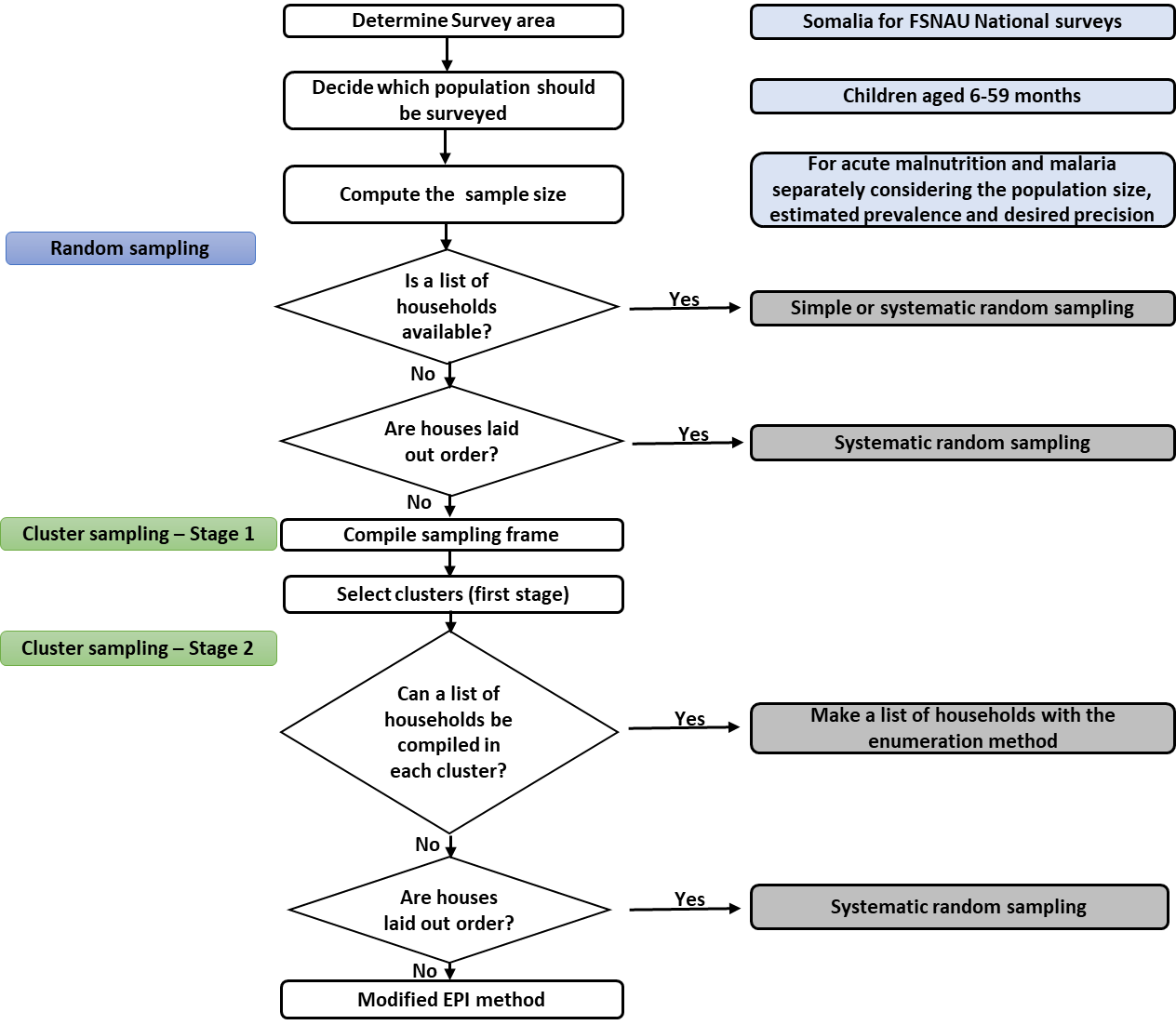


**Figure SI 1:** Flowchart for FSNAU surveys. This diagram was adopted from the ‘Guidelines for emergency nutrition and mortality surveys in Somalia’. The sample size of acute malnutrition and malaria are computed separately depending on the estimated prevalence and the desired precision but the sampling procedure is the same.

**
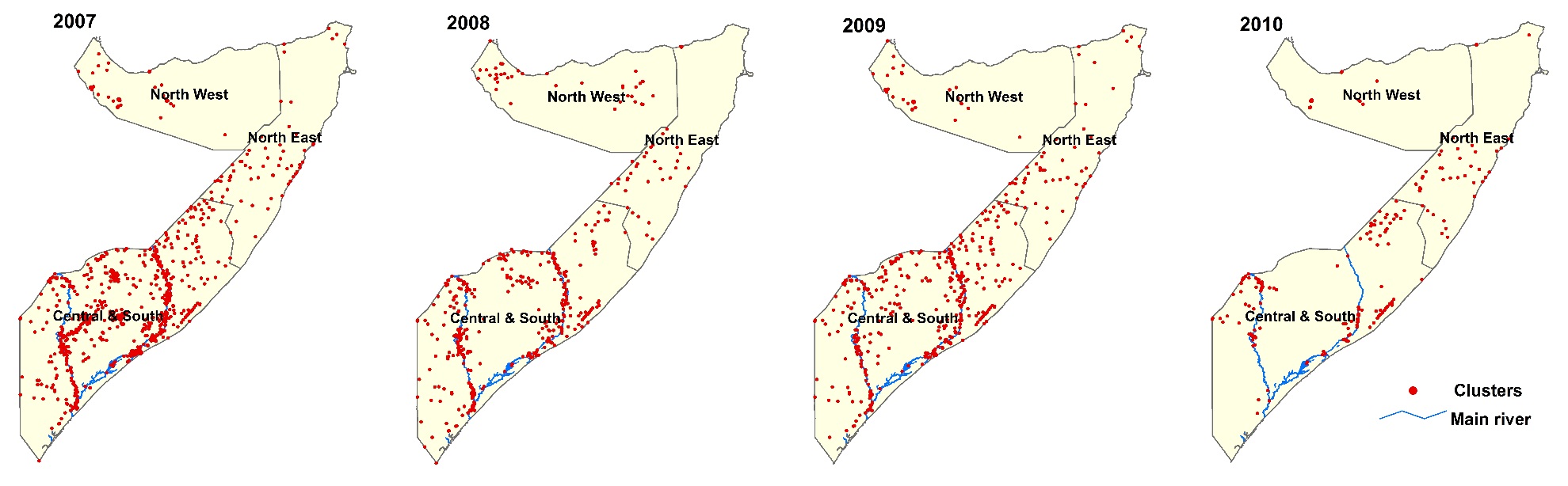
**

**Figure SI 2:** Patterns of stunting among children under the age of five in Somalia. These data were obtained from Food Security and Nutrition Unit (FSNAU) surveys ranging from the year 2007 to 2010.
